# Mechanism underlying the action of Duanteng-Yimu Tang in regulating Treg/Th17 imbalance and anti-rheumatoid arthritis

**DOI:** 10.1016/j.heliyon.2023.e15867

**Published:** 2023-04-29

**Authors:** Wei Feng, Xin Wan, Shirong Fan, Cui-Zhen Liu, Xue-Xia Zheng, Qing-Ping Liu, Min-Ying Liu, Xiao-Bao Liu, Chang-Song Lin, Li-juan Zhang, De-tang Li, Qiang Xu

**Affiliations:** a The First Clinical Medicine School, Guangzhou University of Chinese Medicine. Guangzhou 510405, China; b Department of Rheumatology, The First Affiliated Hospital of Guangzhou University of Chinese Medicine. Guangzhou 510405, China; c Department of Otorhinolaryngology, Zhongshan Hospital Affiliated to Guangzhou University of Chinese Medicine, Zhongshan, China; d Department of Pharmacy, The First Affiliated Hospital of Guangzhou University of Chinese Medicine, Guangzhou, China

**Keywords:** Rheumatoid arthritis, Duanteng-Yimu Tang, Th17/Treg, Balance, Inflammatory factors

## Abstract

**Background:**

Rheumatoid arthritis (RA) is a chronic immune disease characterised by synovitis and cartilage destruction. Currently, many patients experience poor remission after new antirheumatic drug treatments. Duanteng-Yimu Tang (DTYMT), a traditional Chinese medicine, is effective in the treatment of RA. In this research, we designed to investigate the anti-RA effects of DTYMT and explore its potential mechanisms.

**Methods:**

Network pharmacology was adopted to explore the main pathways of DTYMT in patients with RA. Collagen-induced arthritis models of male DBA/1 mice were established, and their histopathological changes were observed by hematoxylin-eosin staining and micro-CT. qRT-PCR was performed to detect the expression of Foxp3 and RORγt in the serum and synovial tissue and IL-17, IL-1β, TNF-α, and IL-10 mRNA in vivo. The proliferation and invasion of synovial cells were analyzed using Cell Counting Kit-8 and transwell assays, respectively. The ratio of T helper 17 (Th17) to regulatory T (Treg) cells was analyzed by flow cytometry.

**Results:**

Network pharmacology analysis revealed that Th17 cell differentiation may be the key pathway of DTYMT in RA. DTYMT ameliorated joint damage, inhibited RORγt expression, and increased Foxp3 expression in CIA mice. DTYMT significantly decreased IL-1β, IL-17, and TNF-α mRNA levels, and increased IL-10 mRNA levels in IL-6-induced cells. Additionally, DTYMT inhibited Th17 cell differentiation and promoted Treg cell production, thus improving the Treg/Th17 imbalance. DTYMT also inhibited the proliferation, migration, and invasion of RA fibroblast-like synovial cells.

**Conclusions:**

These results indicate that DTYMT could regulate the Treg/Th17 cell balance, which is a possible mechanism of DTYMT in treating RA.

## Highlights

• Traditional Chinese medicine is effective for treating rheumatoid arthritis.
• Treg/Th17 cells play an important role in rheumatoid arthritis.
• DTYMT may treat rheumatoid arthritis by regulating Treg/Th17 cell ratio.

## Graphical abstract

**Figure:**
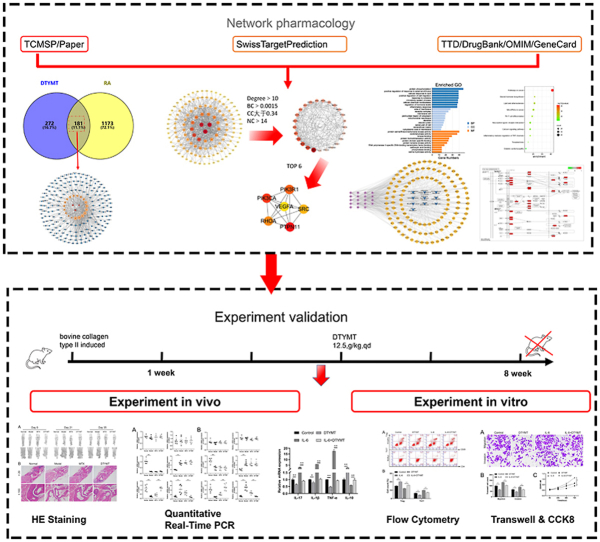

## 1 Introduction

Rheumatoid arthritis (RA) is a systemic autoimmune disease characterised by synovitis and extra-articular organ involvement, such as interstitial pneumonia, and clinical symptoms such as pain, swelling, multiple joint stiffness, and fever [1]. Synovitis is caused by the synthesis of significant quantities of pro-inflammatory factors such as tumour necrosis factor (TNF), interleukin (IL)-1, and IL-6 by lymphocytes and synovial cells in synovitis lesions [2]. Irreversible bone and joint deterioration can start quickly after the onset of the disease, increasing the risk of significant infection and other complications [3,4].

Th17 cells, the principal producers of IL-17, are essential for the pathogenesis of most common autoimmune disorders [5]. In RA, IL-17 enhances synovial cells aggression and stimulates the expression of other pro-inflammatory factors, causing cartilage and bone destruction [6]. Conversely, regulatory T (Treg) cells are important in suppressing autoimmune disease processes [7]. Consequently, restoring homeostasis of Th17/Treg cells is particularly important in RA [8]. Multiple studies have demonstrated that RA patients had higher levels of Th17 cells and their cytokines than normal participants. Niu et al. [9] revealed that an dysregulation of Th17/Treg cell due to increased peripheral blood Th17 cells and decreased Treg cells in individuals with RA may have crucial contribution to the development of RA. In mouse models of RA, improving Foxp3 expression and restoring Th17/Treg balance decreased the progression of arthritis [10,11].

Traditional Chinese medicine (TCM) has long-term practice in treating RA with significant efficacy and few side effects. In RA, reducing proliferative capacity of RA fibroblast-like synoviocytes (RA-FLS) is key to the therapy of RA. Lee et al. [12] found that *Angelica* ethyl acetate inhibits IL-6β-induced proliferation and inflammatory responses in rheumatoid synovial fibroblasts, and Zhai et al. confirmed that imperatorin may promote FLS apoptosis through mitochondrial/caspase-mediated pathways [13]. Li et al. [14] reported that tetrandrine has immunosuppressive and anti-inflammatory properties and alleviates the pathological manifestations of RA in rats. Jia et al. [15] observed that tetrandrine significantly reduced bone destruction and the amount of osteoclasts in CIA rats. These findings suggest that TCM has enormous potential in the treatment of RA.

Duanteng-Yimu Tang (DTYMT) is a small-compound prescription widely used by our team for the treatment of RA. It comprises *Tripterygium hypo-glaucum* (H. Lév.) Hutch (30 g), *Leonurus japonicus* Houtt. (30 g) and *Dipsacus asperoides* C. Y. Cheng et T. M. Ai (15 g). Our previous study showed that DTYMT could improve bone metabolism indicators in RA individuals, such as procollagen I N-terminal propeptide and C-terminal telopeptide of type I collagen (β-CTX) [16]. Leonurine is a unique alkaloid isolated from Herba Leonuri and is a major compound of DTYMT. We verified that leonurine effectively alleviated synovitis and joint degeneration in CIA mice [17]. Network pharmacology is an efficient method for clarifying the modes of action of Chinese herbs and prescriptions. In this research, we collected the targets of DTYMT and identified related pathways which may be the underlying mechanism for DTYMT in RA.

The involvement between Th17/Treg imbalance and RA, as well as the possible mechanisms of TCM in the therapy of RA, are still not fully elucidated. Therefore, this study focused on the mechanism of Chinese medicine associated with the homeostasis of Th17/Treg cells in RA to better exploit the advantageous role of TCM in RA or other refractory diseases.

## 2 Materials and methods

### 2.1 Collection of DTYMT targets

DTYMT compounds were sourced from the Traditional Chinese Medicine Database and Analysis Platform (TCMSP) (https://tcmsp-e.com/) [18] and complemented from papers in Chinese National Knowledge Infrastructure (CNKI) (http://www.cnki.net/), Weipu database (http://www.cqvip.com), and Wangfang database (http://wanfangdata.com.cn/index.html). The compounds were screened based on bioavailability (OB) ≥ 30%, drug likeness (DL) ≥ 0.18 [19], and were converted into Canonical SMILES in Pubchem (hhtp://pubchem.ncbi.nlm.nih.gov). Then, DTYMT related targets from SwissTargetPrediction with the species limited to “Homo sapiens” (Probability＞0.1) were screened.

### 2.2 Screening of RA targets

RA-related targets were obtained from The Online Mendelian Inheritance in Man (OMIM) (http://omim.org/), the Therapeutic Targets Database (TTD) [20] (http://bidd.nus.edu.sg/group/cjttd/), the DrugBank (http://go.drugbank.com/), and the human gene database GeneCards (http://genecards.org/). The compounds and disease overlap proteins were identified as potential RA therapeutic targets.

### 2.3 Network and protein-protein interaction analysis

The PPI analysis was performed by STRING with “Homo sapiens” as the species selection, and 0.9 was set as the lowest interaction score. Then, the visualizing network was constructed through Cytoscape 3.9.0 version. Next, the main target was screened based on the degree, closeness centrality (CC), betweenness centrality (BC), and neighborhood connectivity (NC). Finally, used CytoHubba to selected the topological potential of targets.

### 2.4 Enrichment analysis

Imported the obtained gene list (the common target obtained in 2.2.) into the Metascape (https://metascape.org/) to analyze the Gene Ontology (GO) function and Kyoto Encyclopedia of Genes and Genomes (KEGG) pathway enrichment. The species was limited to humans, the *P* value p was set as 0.01, and the min enrichment was set as 1.5. Next, the visualization of GO and KEGG Pathway Enrichment was completed in bioinformatics (http://www.bioinformatics.com.cn/).

### 2.5 Animals

Twenty-four 9-week-old male mice (18.89+-1.93g）, SPF-grade DBA/1, were purchased from the Experimental Animal Center of Guangzhou University of Chinese, and the animal experiments were completed in the Experimental Animal Center of Guangzhou University of Traditional Chinese Medicine. The animals were placed in individually ventilated cages in the IVC system under SPF condition (temperature, 23–27 °C; humidity, 50%–70%; 12 h day/night alternated), and access to food and water freely. The animals were pre-fed for approximately seven days after purchase for acclimatization before conducting the experiments. Primary T cells and fibroblast-like synovial cells were isolated from the whole blood and synovial tissue of mice.

### 2.6 Reagents

Bovine type II collagen, Freund's complete adjuvant, and Freund's incomplete adjuvant were purchased from Chondrex Inc. (Washington, USA). Methotrexate (MTX) was purchased from Puri Pharmaceutical (Shanxi, China). Mir-X™ miRNA First Strand Synthesis Kit, Mir-X™ miRNA qRT-PCR SYBR® Kit were purchased from Clontech (Mountain View, USA). Affinity Script QPCR cDNA Synthesis Kit, and Brilliant II SYBR Green QPCR Master Mix Kit were purchased from Agilent (Santa Clara, USA). Human IL-6 was purchased from PEPROTECH. Inc. (Cranbury, USA). The TCM standard Chlorogenic Acid, Loganin, Rutin, Quercetin and Asperosaponin Ⅵ were purchased from National Institutes for Food and Drug Control of China (Beijing, China). HPLC-grade methanol were purchased from Merck (Merck, Darmstadt, Germany). Phosphoric acid purchased in Tianjin Fuyu Fine Chemical Co., Ltd. (Tianjin, China). Water is purchased from Watsons. (Guangdong, China).

### 2.7 Preparation of medicine

DTYMT was composed of Tripterygium hypoglaucum (H.Lév.) Hutch (30 g) (batch No. 210701, Guangxi, China), Leonurus japonicus Houtt. [Lamiaceae] (30 g) (batch No. 210901, Guangdong, China) and Dipsacus asperoides C. Y. Cheng et T. M. Ai (15g) (batch No. X0421512, Sichuan, China) (Table 1). All the herbs sourced from the first Affiliated Hospital of Guangzhou University of Chinese Medicine. 10 times the amount of water in the whole formulation was add, the Tripterygium hypoglaucum was firstly boiled for 3.5 h, then mixed two other herbs together and allowed by boiled for 0.5h, and filter liquid; Next, extract the Tripterygium hypoglaucum, Leonurus japonicus, Dipsacus asperoides for 0.5 h, filter and combine filtrate twice. Then the extract was concentrated to 1 g/mL and frozen storage at −20 °C for further analysis.

**Table 1:** Components of DTYMT.

Latin name	Family	English name	Chinese name	Grams (g)	Used part
Tripterygium hypoglaucum (Levl.) Hutch	Celastraceae	Tripterygium hypoglaucum radix	Kunming shanhai tang	30	Root
Leonurus japonicus Houtt	Lamiaceae	Motherwort	Yimu cao	30	Whole plant
Dipsacus asperoides C. Y. Cheng et T. M. Ai	Dipsacus L	Dipsaci Radix	Xuduan	15	Root

MTX powder was dissolved completely by adding 550 μL PBS to each vial of MTX to obtain a MTX concentration of 20 mmol/L (mM). The MTX was then dispensed in sterile EP tubes at 100 μL per tube in an ultraclean bench and stored at −80 °C.

### 2.8 High performance liquid chromatography (HPLC) analysis for DYYMT

HPLC was utilized to identify the potential active substances of DTYMT. In brief, 10 mL DTYMT dilution solution (DTYMT: water = 1:9) and stands were respectively injected into the 1260 Infinity II High Performance Liquid Chromatograph equipped with a diode array detector working in the range of 200–400 nm, column temperature chamber and autosampler (Agilent Technology, CA, USA). A Kromasil 100-5-C18 column (4.6 mm*250 mm, 5 μm, Nouryon, Sweden）was used for the separation of the components and the flow column temperature was 30 °C. The mobile phase consisted of methanol (A) and 0.02% phosphoric acid water (B). The gradient elution programme was used as follows: 0–5 min,15–20%A; 5–30 min, 20–48%A; 30–40 min, 48–57%A; 40–45 min, 57-36%A; 45–55 min, 78–80%A and the re-equilibration time of gradient elution was 5 min. The flow rate was 1 mL/min, and the injection volume was set at 10 μL. Peaks were monitored at 224 nm.

### 2.9 Establishment of the CIA model

Each mouse except the normal the group was injected intradermally with 100 μl of emulsified CII/Freund's adjuvant (CFA) (on day 1), and was given a booster immunization of CII mixed incomplete Freund's adjuvant (IFA) on day 21 to establish CIA model [21].

### 2.10 Experimental design

Based on our previous study, 12.5 g/kg was selected as the DTYMT group [22]. The animals were randomized into four groups with a random number table (n = 6, mean = 18.56g, SD = 1.74g): normal group (Normal), model group (Model), methotrexate group (MTX), and Duanteng-Yimu Tang group (DTYMT). Each group was given the corresponding treatment at the second immunization of the CIA mouse model, i.e., on day 21 of modeling (the day the modeling was completed). The mice were treated with DTYMT(12.5 g/kg daily), MTX (2 mg/kg twice a week) or identical volume of saline by gavage for two weeks [23], followed by decervicalization and execution on day 35. Blood, synovial membrane, and cartilage tissues were collected on day 35.

### 2.11 HE staining of mouse ankle joints

Mice ankles were placed in 4% paraformaldehyde solution for 24 h. They were then placed in 10% EDTA for decalcification at 37 °C for 15 days, followed by gradient dehydration in PBS containing 20% and 30% sucrose. The tissues were sagittally sectioned at a thickness of 15 μm in the downstream direction using a frozen sectioning machine at −20 °C. The sections were directly attached to gelatin-treated slides and then air-dried. HE staining of the weaves was performed as follows: sections were first rinsed with water for 1–2 s, stained with hematoxylin for half a minute, rinsed with water for 5–10 s, rinsed with 1% hydrochloric acid ethanol fractionation solution for 1–3 s, rinsed with running water for 5–10 min, stained with eosin for 10 s, rinsed with water for 1–2 s, rinsed with 80% ethanol for 1–2 s, rinsed with 95% ethanol for 1–2 s, rinsed with anhydrous ethanol rinse for 1–2 s, and rinsed with xylene for 1–3 s. Finally, the woven sections were sealed with neutral gum. Stained sections were observed and photographed under a microscope.

### 2.12 Serum and PBMC cells isolation

The top serum component of the obtained mouse whole blood was aspirated after centrifugation at 3000 rpm for 10 min. To remove cellular debris and platelets, the serum was centrifuged at 3000 rpm for 15 min. Serum was then obtained from the patient. After complete mixing, the supernatant was transferred to a fresh sterile EP tube, placed on ice, and incubated for 30 min with Total Exosome Isolation reagent at a 5:1 vol ratio (clouding will occur). The PBMC cells were resuspended in 100 L of PBS and stored at −80 °C in a refrigerator for evaluation.

### 2.13 Quantitative real-time PCR (qRT-PCR)

For the Foxp3 and RORγt quantitative detection, the required reagents were added according to the instructions of the Brilliant II SYBR Green QPCR Master Mix Kit. The primers for Foxp3, RORγt, and β-actin were as follows: Foxp3, forward 5ʹ- TCTCCAGGACAGACCACACT -3ʹ and reverse 5ʹ- CAGCAGAAGGTGGTGGGAG-3ʹ; RORγt3, forward 5ʹ- ACCAGGCATCCʹ and reverse 5ʹ- GCATATGC-3; β-actin, forward 5ʹ- AGGGAAATCGTGCGTGACAT -3ʹ and reverse 5′- GAACCGCTCATTGCCGATAG-3ʹ. The PCR reaction program was conducted as follows: 95 °C for 5 min; 95 °C for 5 s; 60 °C for 20 s; cycle 38 times, and finally stored at 4 °C. All the reaction experiments were repeated three times. β-actin was used as the internal control for Foxp3 and RORγt to analyze the Ct value of PCR amplification between Foxp3 and RORγt internal control β-actin. 2^−ΔΔCt^ method was adopted for calculation.

### 2.14 Cellular experimental grouping and drug administration

Mouse primary T cells were stimulated with IL-6 (30 ng/mL) and treated with DTYMT (600 μg/mL). The experiment was divided into four groups: control, DTYMT, IL-6, and IL-6+DTYMT groups. After 48 h of culture, qRT-PCR was performed to detect the expression of IL-17, IL-1β, TNF-α, and IL-10 in each group of cells. CCK8 was used to detect the proliferation of synovial cells, while Transwell was adopted to measure the migration and invasion of synovial cells. Anti-CD4 antibody (2 μg/mL) and anti-CD25 antibody (2 μg/mL), and the ratio of Th17 and Treg cells were analyzed by flow cytometry.

### 2.15 Statistical analysis

SPSS version 19.0 (SPSS Inc., Chicago, IL, USA) was conducted for data analysis. One-way analysis of variance was used to compare the means between groups, and statistical significance was set at P < 0.05. Independent two-group analyses was conducted by the Student's t-test. The data are expressed as the mean ± standard deviation base on ≥ triple repeat (n = 6 per group).

## 3 Results

### 3.1 Targets between DTYMT and RA

**Fig. 1:**
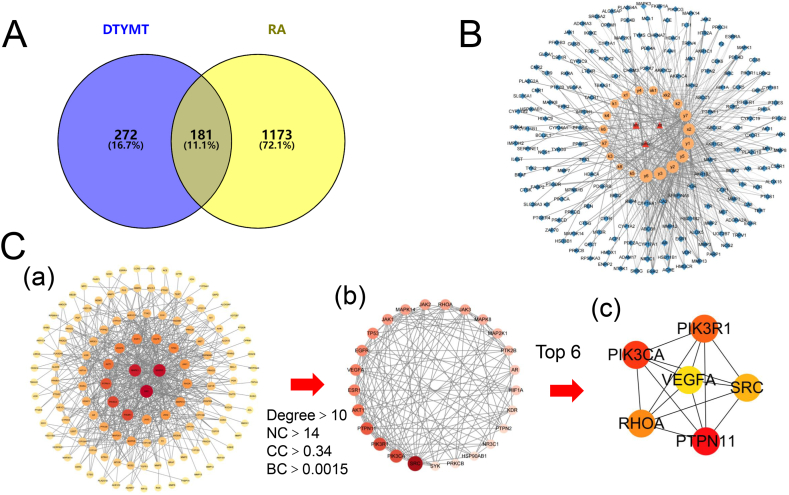
Network of Targets between DTYMT and RA. (A) Venny picture of DTYMT and RA. (B) Network of overlapping targets. 181 targets were Blue diamond, compounds of DTYMT were orange round, and herbs were red triangle. (C): (a) The PPI network of targets. (b) According to the scores of CC, BC, NC and degree. (c) The top 6 were core genes. (For interpretation of the references to colour in this figure legend, the reader is referred to the Web version of this article.)

A total of 33 candidate compounds were obtained using TCMSP supplemented with papers. After excluding invalid compounds, which could not be found in the canonical SMILES in Pubchem or had related target probabilities lower than 0.1 based on the SwissTargetPrediction result, 19 compounds of DTYMT were selected, 453 targets were obtained, and 1354 targets were identified for RA. There were 181 targets overlapped between DTYMT and RA according to the Venny analysis (Fig. 1A). The DTYMT compounds-targets network is visualized in Fig. 1B, including 203 nodes and 540 edges.

### 3.2 PPI network analysis

The PPI network consisted of 132 nodes and 533 edges (Fig. 1C–a), and targets higher than the median of BC, CC, and NC (0.0015, 0.340, and 14, respectively) and twice the medians node degree (10) were considered for the subsequent analysis. Finally, 25 targets were obtained (Fig. 1C–b), and the top six targets calculated by CytoHubba with the MCC algorithm were PIK3CA, PHOA, VEGFA, PIK3R1, PTPN11, and SRC (Fig. 1C–c). These may be core targets for treating RA.

### 3.3 Gene ontology analysis and KEGG pathway enrichment analysis

**Fig. 2:**
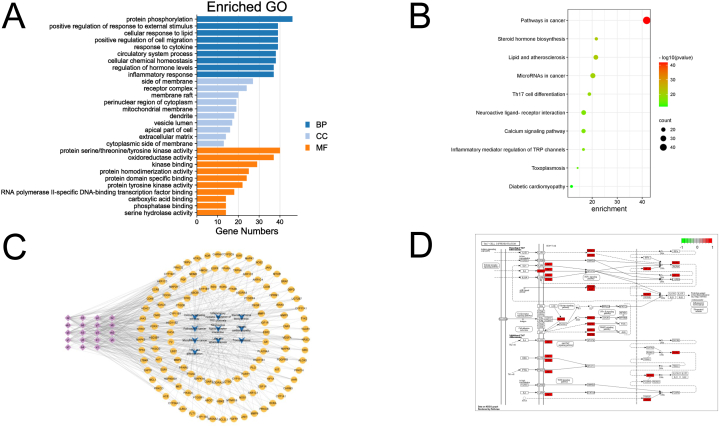
KEGG and GO pathway analysis. (A) GO enrichment analysis. (B) The KEGG pathways. (C) Network of pathway-target-compound, including the pathway (bule “V”), the targets (yellow round), and the Chinese medicine in DTYMT (purple diamond). (D) The visualization of the Th17 cell differentiation pathway. (For interpretation of the references to colour in this figure legend, the reader is referred to the Web version of this article.)

A total of 181 targets between DTYMT and RA were analyzed. Using gene ontology (GO) enrichment analysis, 2131 GO items were collected, including 96 CC, 1804 BP, and 231 MF. The top 10 enriched CCs, BPs, and MFs were visualized in Fig. 2A. To further elucidate the association between candidate targets and their pathways, the top 20 pathways were displayed (Fig. 2B and C). The main terms included pathways in cancer, steroid hormone biosynthesis, lipid and atherosclerosis, microRNAs in cancer, and Th17 differentiation. The Th17 differentiation pathway was enriched by MAPK14, MTOR, HIF1A, HSP90AB1, IL6ST, JAK1, JAK2, JAK3, PRKCQ, MAPK1, MAPK3, MAPK8, RORC, RXRA, TYK2, and ZAP70 (Fig. 2D).

### 3.4 Analysis of the potential active ingredient of DTYMT

**Fig. 3:**
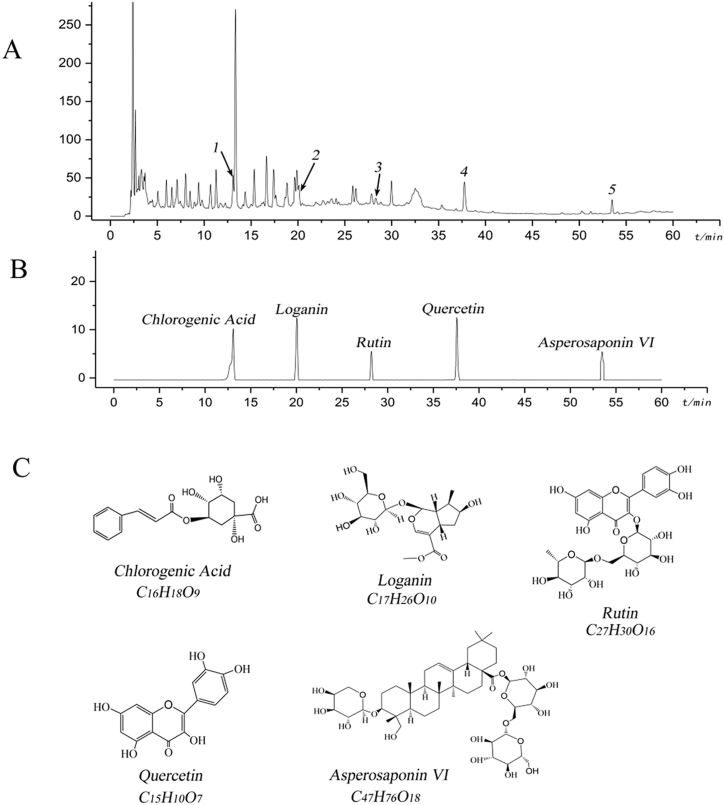
HPLC analysis of the chemical components in the DTYMT decoction. (A) HPLC fingerprint of DTYMT decoction. (B) HPLC chromatogram of standard reference. (C) Chemical formulas and equations for Chlorogenic Acid, Loganin, Rutin, Quercetin and Asperosaponin Ⅵ. The experiments were performed in triplicate.

Analyses were performed using Agilent Chromatography workstation software (Agilent Technology, CA, USA). To elucidate the chemical components, unknown compounds were identified by comparison with the standard reference data, and five compounds were identified in the DTYMT extract, namely chlorogenic acid, loganin, rutin, quercetin, and asperosaponin VI. The high performance liquid chromatography chromatograms of DTYMT and the reference substance are shown in Fig. 3A–C.

### 3.5 Effects of DTYMT in joints of CIA mice

**Fig. 4:**
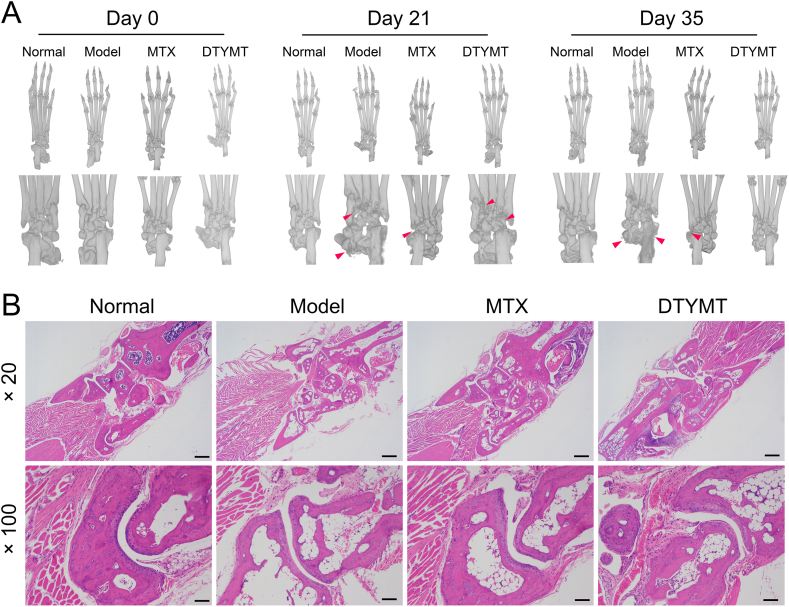
Effects of DTYMT on the ankle joint in CIA mice. (A) Micro-CT scan of ankle bone in CIA mice. From left to right, days 0, 21, and 35 are shown in order. (B) HE staining of the ankle joint of mice on day 35 ( × 20, scale bar = 500 μm; × 100, scale bar = 100 m). n = 6 per group.

CIA models were used to assess RA treatment with DTYMT. As shown in Fig. 4A, CIA mice began to exhibit bone damage on day 21. On day 35, the joints in the CIA model displayed severe bone loss and an incomplete structure. According to the micro-CT scan, the mice in the MTX and DTYMT groups had reasonably undamaged bones and less bone loss than the model group. The HE staining results (Fig. 4B) displayed that the mice ankles treated with MTX and DTYMT were relatively intact, with insignificant synovial thickening, a limited amount of infiltrated inflammatory cells, and relatively less articular cartilage and bone destruction. In general, the efficacy of DTYMT was superior to those of MTX.

### 3.6 Effects of DTYMT on the expression levels of Foxp3 and RORγt mRNA in PBMC and synovial tissues of CIA mice on day 35

**Fig. 5:**
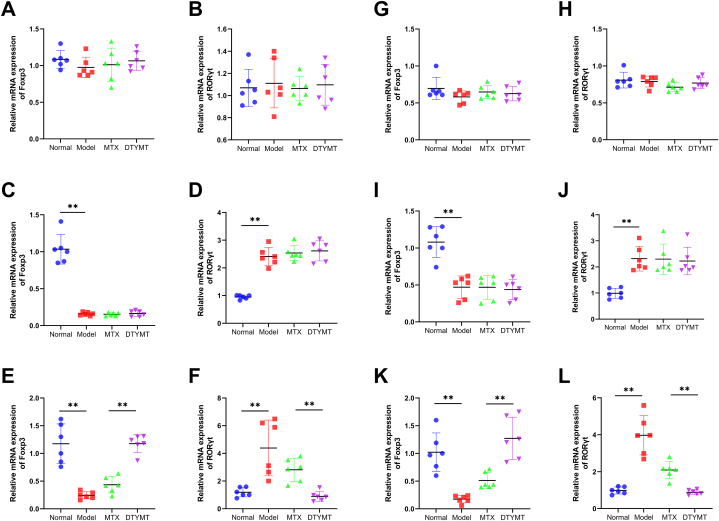
qRT-PCR of Foxp3 and RORγt in CIA mice. (A–F) The expression of Foxp3 and RORγt in PBMC. The top, middle, and bottom pictures were at days 0, 21, and 35, respectively. (G–J) The expression of Foxp3 and RORγt in synovial tissue. The top, middle, and bottom pictures were at days 0, 21, and 35, respectively. n = 6 per group. The experiments were performed in triplicate. *P < 0.05, **P < 0.01.

We used qPCR to analyze the expression of Foxp3 and RORγt in mice. Fig. 2A-B, G-H showed the expression of Foxp3 and ROR**γ**t in mice on Day 0. On day 21, Foxp3 expression was evidently inhibited, while the expression of RORγt was obviously increased in both PBMCs (Fig. 5C and D) and synovium (Fig. 5I and J), which was also observed in the model group on day 35 (Fig. 5E–F, K-L). DTYMT clearly increased Foxp3 levels and inhibited the expression of ROR**γ**t in both PBMCs (Fig. 5E and F) and synovial tissues (Fig. 5K-L), relative to the model group on day 35. In addition, the effects of DTYMT were better than those of MTX alone. These results suggest that DTYMT can effectively alleviate joint damage in CIA mice and may be involved in Th17 differentiation.

### 3.7 Effect of DTYMT on the mRNA expression levels of IL-17, IL-1β, TNF-α, and IL-10 in primary T cells

**Fig. 6:**
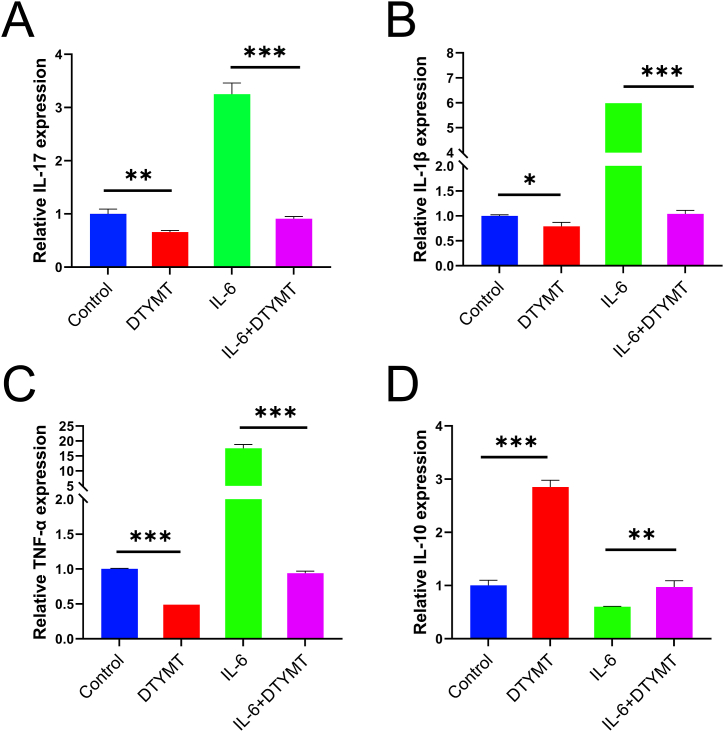
Inflammatory factor expression. qRT-PCR of IL-17, IL-1β, TNF-α, and IL-10 in Primary T cells. IL-6+DTYMT group was stimulated by IL-6 for 24h followed by de-intervention with DTYMT for 24h. *P < 0.05, **P < 0.01, and ***P < 0.001. n = 3 per group. The experiments were performed in triplicate.

IL-6 stimulation (30 ng/mL) was administered with T cells for 24 h before DTYMT was administered as a pharmacological intervention for 24 h. The cytokines (IL-17, IL-1, TNF-, and IL-10) mRNA levels in T cells were measured applying RT-qPCR assay. DTYMT significantly downregulated the expression of IL-17, IL-1β, and TNF-α but upregulated IL-10 expression in primary T. The levels of IL-17, IL-1β, and TNF-α were substantially lower in the IL-6 + DTYMT group than in the IL-6 alone group, while the IL-10 mRNA expression level was significantly higher. The above findings indicated that DTYMT inhibited the production of IL-17, IL-1β, and TNF-α mRNA while boosting IL-10 expression in primary T cells. (Fig. 6A–D).

### 3.8 Effect of DTYMT on the proportion of Th17 cells and treg cells differentiated from initial T cells

**Fig. 7:**
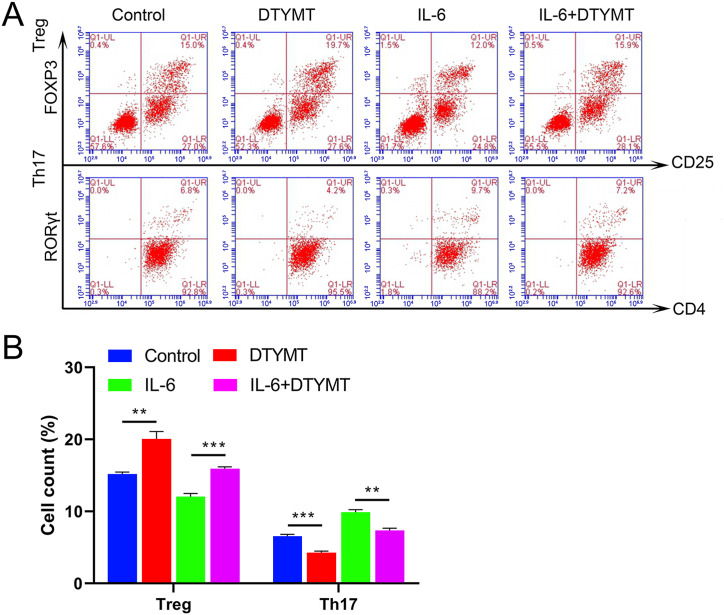
Proportion of Th17 and Treg cells differentiated by primary T cells. (A) Flow cytometry detection of Treg and Th17 cell ratios in each group. (B) Histogram of the number of Th17 and Treg cells in each group (n = 3). **P < 0.01, and ***P < 0.001. The experiments were performed in triplicate.

Anti-CD4 and anti-RORt antibodies were adopted to mark Th17 cells, whereas Treg cells were labelled with anti-CD25 and anti-Foxp3 antibodies. Flow cytometry was employed to determine the ratio of Th17 to Treg cells. The amount of Th17 cells was obviously diminished, while the amount of Treg cells was substantially enlarged after DTYMT drug intervention in relation to the control group (Fig. 7A). The proportion of Th17 cells was descended after IL-6 stimulation followed by DTYMT drug intervention in contrast to that in the IL-6 group, while the proportion of Treg cells was considerably elevated (Fig. 7B).

### 3.9 Effect of DTYMT on cell migration and invasion after initial T cell co-culture with synovial cells

**Fig. 8:**
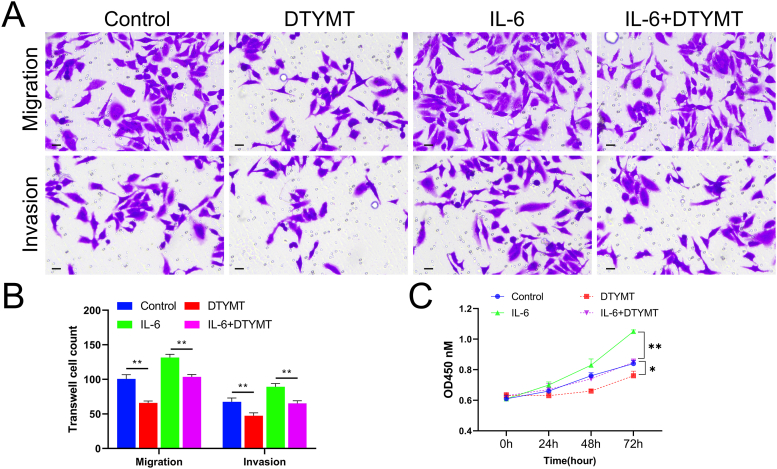
Cell invasion and migration after co-culturing of primary T cells and CIA mouse synovial cells. (A) Transwell assays for cell invasion and migration in each group ( × 200, scale bar = 50 μm, n = 3). (B) Columns of transwell assays. (C) CCK8 detected the proliferation ability of each group of cells. *P < 0.05, **P < 0.01. The experiments were performed in triplicate.

IL-6-stimulated T cells were co-cultured with RA fibroblast -like synoviocytes (RA-FLS), and then DTYMT was added for 48 h. Transwell assays revealed that there were much smaller numbers of migrating and invading cells in the DTYMT group than those in the control group. The amount of migrating and invading RA fibroblastic synovial cells was considerably lower by DTYMT addition than IL-6 treatment alone. Cell migration and invasion abilities dramatically decreased (Fig. 8A and B). The above results indicate that DTYMT considerably weakened the migratory and invasive capacity of RA fibroblastic synovial cells under normal conditions or after co-culture with IL-6-stimulated T cells.

### 3.10 Effect of DTYMT on cell proliferation after Co-culture of initial T cells and RA synovial cells

T cells were pre-treated with IL-6 and co-cultured with RA-FLS for 48 h. Subsequently, we added DTYMT for intervention, and after 48 h, we used the CCK8 technique to detect the effect of RA-FLS growth. The proliferation capacity of DTYMT cells was substantially descended in relation to that of the control group, and the proliferation ability of RA-FLS in the IL-6+DTYMT group was prominently suppressed in contrast to that of the IL-6 group (Fig. 8C). These findings revealed that DTYMT substantially suppressed the proliferation of RA-FLS under normal conditions and the proliferation of T cells co-cultured with RA-FLS after IL-6 stimulation.

## 4 Discussion

RA is typified by synovial inflammation and cartilage damage, leading to joint disability [24]. The imbalance in Th17/Treg cells is crucial in the pathogenesis of RA synovitis [25]. Currently, medications such as MTX and TNF have some limitations in RA treatment, which emphasises the need for novel drug discovery [26]. This research confirmed that DTYMT significantly alleviated inflammation and damage to the affected joints in CIA mice. In addition, DTYMT increased Foxp3 expression and inhibited RORγt expression in the CIA model. Furthermore, DTYMT had a significant suppression on the proliferation and migration of FLS and restored Th17/Treg cell homeostasis in an in vitro model. These results indicated that DTYMT may treat RA by mediating the Th17/Treg cells homeostasis.

In the current study, 19 active components of DTYMT were screened and 181 possible targets for DTYMT in RA were predicted. Network pharmacology analysis demonstrated that DTYMT appeared to treat RA through multiple pathways and targets. KEGG enrichment analyses suggested that Th17 cell differentiation pathway might be involved in the treatment of DTYMT on RA. Th17 cells are drivers in several autoimmune diseases, and autoimmune diseases can be effectively treated by correcting the abnormal proliferation of Th17 cells, especially in RA [27]. Genes, including JAK1, were enriched in the Th17 cell differentiation pathway. The JAK-related pathways are responsible for cell proliferation, differentiation, apoptosis, and immune regulation. JAK maintains an inflammatory state by regulating various signals [28].

Our laboratory has previously demonstrated that DTYMT significantly improves joint swelling, joint pathology, and cartilage destruction in mice with CIA. This indicates that DTYMT could significantly treat mice with RA, but the mechanism remains largely unknown and needs to be investigated further. In our study, the ankle joints of mice in the MTX and DTYMT groups showed no significant redness or swelling, and joint mobility was not substantially different from that of normal mice. The effect of DTYMT in treating CIA mice was considerable and equivalent to that of methotrexate. HE staining demonstrated that the ankle joint tissues of the normal group were structurally intact, with no inflammatory cell infiltration or hyperplastic synovium. No articular cartilage or bone damage was observed, and the articular cartilage surfaces were smooth and flat. The ankles of the model group were severely injured, with evidence of inflammatory cell infiltration and synovial cell proliferation, resulting in cartilage and bone degradation. The ankles of the DTYMT and MTX groups were unaffected, with little synovial membrane thickening or inflammatory cell infiltration. DTYMT prevents RA development in mice.

Foxp3 is a critical transcription factor for Tregs and plays an essential role to maintain their immunosuppressive function. Treg cell dysfunction occurs in the RA synovium, and increased IL-6 disrupt Treg cell homeostasis and function and accelerate the advancement of pro-inflammatory T cell subsets [29,30]. RORγt is a species isoform transcribed from the RORC gene and is expressed in thymocytes, Th17 cells, and Tc17 [31]. RORγt is regarded as an important pathway for Th17 differentiation through medicating Th17 genes expression and producing IL-17A, IL-17F, and IL-22 in innate and adaptive immune cells [32,33] Previous studies confirmed that IL17/RORγt signalling inhibition could suppress Th17 cell differentiation and protect joints in CIA mice [34,35]. Our study revealed that DTYMT and MTX upregulated Foxp3 expression while downregulating RORγt expression in CIA mice, with DTYMT having a stronger effect than MTX. It is possible that DTYMT treats CIA mice by raising the amount of Treg cells Treg cells in their PBMCs and synovial tissue while lowering the number of Th17 cells. In vitro experiments also demonstrated that DTYMT enhanced the mRNA levels of Foxp3 and decreased RORt expression in RA fibroblastic synovial cells under normal conditions or after IL-6 stimulation. IL-6 evidently stimulated the growth of Th17 cells and decreased the number of Treg cells. However, DTYMT increased the proportion of Treg cells and downregulated the proportion of Th17 cells, bringing Treg and Th17 cells closer to normal levels. It was verified in vitro that DTYMT might perform an ameliorative effect on CIA mice via restoring the dysregulation of Th17/Treg cells in blood PBMCs and synovial tissues.

IL-17, IL-1β, and TNF-α play critical roles in RA development and progression. They cause systemic or local inflammation, which destroys bone and induces pain by activating osteoclasts or inhibiting regulatory T cells [36,37,38,39,40,41]. Our data supported that DTYMT appears to have excellent anti-inflammatory effects. In vitro cellular assays showed that DTYMT inhibited the release of IL-17, IL-1β, and TNF-α in initial T cells, but promoted IL-10 expression. Furthermore, IL-6 stimulation promoted the development of these pro-inflammatory factors in initial T cells but inhibited IL-10 expression. The addition of DTYMT reversed the influence of IL-6 on initial T cells. Consequently, DTYMT might exert anti-inflammatory activities in RA by modulating inflammatory factors release and Th17/Treg cell balance.

The number of FLS and T cells in the synovium of individuals with early RA are immunohistological indicators of poor disease prognosis, which suggests that these cells play significant influence in the process of joint deterioration [42]. Therefore, decreased synovial hyperplasia may be an effective treatment for RA [43]. Here, we verified that DTYMT dramatically reduced the ability of RA fibroblast-like synoviocytes to proliferate under normal circumstances as well as the ability of T cells to proliferate following co-culture with RA fibroblastic synovial cells after IL-6 activation.

Our study has some limitations. First, owing to funding constraints, the exact mechanism by which DTYMT restored the balance of Th17/Treg cells was not explored, and no in-depth verification (including other pathways) was performed. Second, although we used this dose in reference to our previous study, we were unable to demonstrate the effects of other doses. Therefore, we will overcome these limitations in future studies.

## 5 Conclusion

In general, we confirmed that DTYMT alleviated the inflammatory response of CIA mice and medicated the dysregulation of Th17/Treg cells, thereby exerting effective treatment on RA. But it needs further experiments to explore multiple mechanisms of DTYMT in the treatment of RA.

## Author contribution statement

Li-juan Zhang, De-tang Li, Qiang Xu: Conceived and designed the experiments.

Wei Feng, Xin Wan, Shirong Fan: Performed the experiments; Wrote the paper.

Cui-Zhen Liu, Xue-Xia Zheng, Qing-Ping Liu: Analyzed and interpreted the data.

Min-Ying Liu, Xiao-Bao Liu, Chang-Song Lin: Contributed reagents, materials, analysis tools or data.

## Data availability statement

Data will be made available on request

## Institutional review board statement

The study was conducted in accordance with the Declaration of Helsinki, and approved by the Experimental animal ethics committee of Guangzhou University of Traditional Chinese Medicine (animal ethic number 2020006).

## Funding

This study was supported in part by: Natural Science Foundation of Guangdong Province (No. 2019A1515011636), Guangzhou Science And Technology Bureau (No. 201904010177), Grant of the First Affiliated Hospital of Guangzhou University of Chinese Medicine (2019QN08), Guangdong Administration Of Traditional Chinese Medicine (No. 20191110), Department of Education of Guangdong Province (No. 2022KTSCX030). And this work was generously supported by the Guangzhou Science and Technology Project (202201020457), Guangzhou Science and Technology Project (SL2023A03J00683). Medical research fund project of Guangdong Province (No. A2020409), Guangzhou Science and Technology Project, Excellent Young Scholars Project of the First Affiliated Hospital of Guangzhou University of Chinese Medicine (2019QN22). Social public welfare and basic Research project of Zhongshan Science and Technology Bureau(2020B1096).

## Declaration of competing interest

The authors declare that they have no known competing financial interests or personal relationships that could have appeared to influence the work reported in this paper.
